# "Practical knowledge" and perceptions of antibiotics and antibiotic resistance among drugsellers in Tanzanian private drugstores

**DOI:** 10.1186/1471-2334-10-270

**Published:** 2010-09-16

**Authors:** Nina Viberg, Willbrord Kalala, Phare Mujinja, Göran Tomson, Cecilia Stålsby Lundborg

**Affiliations:** 1 Division of Global Health (IHCAR), Department of Public Health Sciences, Karolinska Institutet, Stockholm, Sweden; 2 School of Pharmacy, Muhimbili University of Health and Allied Sciences, Dar Es Salaam, Tanzania; 3 School of Public Health and Social Sciences, Muhimbili University of Health and Allied Sciences, Dar Es Salaam, Tanzania; 4 Medical Management Centre (MMC), Karolinska Institutet, Stockholm, Sweden

## Abstract

**Background:**

Studies indicate that antibiotics are sold against regulation and without prescription in private drugstores in rural Tanzania. The objective of the study was to explore and describe antibiotics sale and dispensing practices and link it to drugseller knowledge and perceptions of antibiotics and antibiotic resistance.

**Methods:**

Exit customers of private drugstores in eight districts were interviewed about the drugstore encounter and drugs bought. Drugsellers filled in a questionnaire with closed- and open-ended questions about antibiotics and resistance. Data were analyzed using mixed quantitative and qualitative methods.

**Results:**

Of 350 interviewed exit customers, 24% had bought antibiotics. Thirty percent had seen a health worker before coming and almost all of these had a prescription. Antibiotics were dispensed mainly for cough, stomachache, genital complaints and diarrhea but not for malaria or headache. Dispensed drugs were assessed as relevant for the symptoms or disease presented in 83% of all cases and 51% for antibiotics specifically. Non-prescribed drugs were assessed as more relevant than the prescribed. The knowledge level of the drugseller was ranked as high or very high by 75% of the respondents. Seventy-five drugsellers from three districts participated. Seventy-nine percent stated that diseases caused by bacteria can be treated with antibiotics but 24% of these also said that antibiotics can be used for treating viral disease. Most (85%) said that STI can be treated with antibiotics while 1% said the same about headache, 4% general weakness and 3% 'all diseases'. Seventy-two percent had heard of antibiotic resistance. When describing what an antibiotic is, the respondents used six different kinds of keywords. Descriptions of what antibiotic resistance is and how it occurs were quite rational from a biomedical point of view with some exceptions. They gave rise to five categories and one theme: *Perceiving antibiotic resistance based on practical experience*.

**Conclusions:**

The drugsellers have considerable "practical knowledge" of antibiotics and a perception of antibiotic resistance based on practical experience. In the process of upgrading private drugstores and formalizing the sale of antibiotics from these outlets in resource-constrained settings, their "practical knowledge" as well as their perceptions must be taken into account in order to attain rational dispensing practices.

## Background

The discovery of antibiotics revolutionized the management of bacterial infectious diseases. Prompt access to and appropriate use of antibiotics render historically lethal diseases curable. Their function is however threatened by growing levels of antibiotic resistance and the problem is especially pertinent in less affluent parts of the world [1-3]. In low-income countries, infectious diseases have great negative impact on public health and use, including over- and misuse, of antibiotics is frequently reported [4-8].

On the other hand, poor people's access to antibiotics is often highly restricted and one third of the world's population is estimated to lack regular access to essential medicines [9,10]. In Tanzania over 90% of the population lives within 10 km of a health care facility.This does however not automatically lead to appropriate drug access since public health care facilities often are in short supply due to an unreliable delivery system [11-13]. Furthermore, private pharmacies are few and located almost exclusively in urban areas [11]. Instead, private drugstores (In Swahili "duka la dawa baridi") licensed to sell over-the-counter drugs (OTC) are alternative sources of medicines for poor people and their importance has been acknowledge by the Ministry of Health [14].

People seek care in private drugstores for mild ailments as well as serious diseases. Some of the main reasons include, but are not limited to, inability to pay both for medical consultation and drugs, proximity of the store to the home, availability of drugs and friendliness of the drugseller [15-18]. However, concerns have been raised about profit aspirations, low quality of practice, insufficient drugseller training and knowledge as well as regulatory infringements in these stores [14,16,19,20]. In an earlier published study from Tanzanian private drugstores, we found that simulated clients were readily sold antibiotics for the treatment of sexually transmitted infection (STI) related complaints in spite of the prescription-only status of these drugs [21]. The opposite was found in a study in Zimbabwean pharmacies [22]. There is a delicate balance between enforcing regulation and maintaining access to important drugs, and resistance development further complicates the issue. In Tanzania efforts are now being made to upgrade the private drugstores into accredited drug dispensing outlets (ADDOs) authorized to sell OTC drugs and a selected range of prescription-only drugs including some antibiotics [13,23].

Perceptions influencing antibiotic dispensing practices by drugsellers are not yet well understood [4]. When searching the literature, no studies on private drugsellers' knowledge and perceptions of antibiotics and resistance could be found. The aim of the present study is to explore and describe antibiotics sale and dispensing practices in private drugstores in rural Tanzania as well as to explore drugseller knowledge and perceptions of antibiotics and antibiotic resistance.

## Methods

### Study setting

This study was conducted in eight districts of Tanzania. Four of Tanzania's 22 mainland regions were purposively selected based on geographical location. Out of these, eight districts were randomly selected, two from each region: Mkuranga and Rufiji (Coast region), Korogwe and Muheza (Tanga region), Mvomero and Kilosa (Morogoro region) and Mpwapwa and Kongwa (Dodoma region). The population size of each district is around 250 000 inhabitants.

A census of drugstores was carried out and a list of all registered drugstores in each district was obtained from the district authority. All drugstores that were still in business at the time of the visits were traced in the eight districts and were included in the study. If found, additional drugstores that were not in the formal list were included during data collection. Ninety-four drugstores were physically identified in the eight districts.

The study was conducted in 2005 within the Peercon project aiming to improve drugstore practice. Exit interviews with drugstore customers were performed in all eight districts at the baseline phase of this project. During the intervention phase consisting of workshops and out-reach visits, interviews with drugsellers were performed before the onset of the actual teaching. Drugseller interviews were conducted in three of the above-mentioned districts that had been randomly allocated to the intervention group while the fourth intervention district had to be excluded due to time constraints. The intervention did not focus on antibiotics specifically but on Good Dispensing Practice (GDP), malaria and STI.

### Data collection

#### Exit interviews with drugstore customers

Consecutive customers from all eligible drugstores in the eight districts were interviewed as they came out of the drugstore. The aim was to interview five customers per store based on power calculations for evaluation of the intervention. The actual number varied between 1-16 customers per store according to feasibility. The customers were asked to show the bought drug and to answer some questions about the encounter such as information provided, satisfaction with the service and perceived knowledge level of the drugseller. They were also asked which disease/symptom they bought drugs for. Data- collectors, mainly pharmacy and medical students, were trained during an interactive workshop led by the Tanzanian co-authors. The data-collectors asked the questions to the customers and filled in the questionnaire, which was developed in English collectively by the research team, translated into Swahili and pretested for clarity. Questionnaire available as additional file 1.

#### Drugseller antibiotic questionnaire

One drugseller from each drugstore identified in the intervention districts was invited to participate in the intervention. The intervention participants were asked to fill in a questionnaire at the onset of either the workshops or the out-reach visits before the actual teaching started. The questionnaire contained closed- and open-ended questions on knowledge and perception of antibiotics, their function and indications as well as the concept of antibiotic resistance. It was developed in English, based on findings from the baseline phase, and translated into Swahili. Pretesting led to some changes being made to the questionnaire mainly concerning the wording. Questionnaire available as additional file 2.

Verbal informed consent was given by the respondents. They were informed that participation was voluntary and confidential and that they could discontinue the study at any point without adverse consequences. It was emphasized that the study was not part of any official drugstore inspection function. Letters of permission were received from district authorities. Ethical approvals for the Peercon project were received through Muhimbili University of Health and Allied Sciences from the Tanzania Commission for Science and Technology No 2003-084-CC-2003-09 and from Karolinska Institutet No 04-514/3.

### Data analysis

Data were analyzed using mixed quantitative and qualitative methods.

#### Analysis of closed-ended questions

The data was entered into Excel, checked and cleaned. The variable "sold antibiotics or not" was selected as an indicator variable for estimating clustering effect over drugstore. Since the clustering effect was very small (ICC = 0.005), individual respondents was used as unit of analysis for the exit-customer interviews. This was also the case for the drugseller interviews. Descriptive analysis was performed and differences in proportions between groups were presented as odds ratios (OR) with 95% confidence intervals (CI) or determined using χ^2 ^-test and, where the groups were too small, Fisher's exact test. A multivariate logistic regression analysis was fitted in Stata 8 using dependent variable: 'knows that antibiotics treat bacterial but not viral disease' and the independent variables: age, sex, education and district. The antibiotics mentioned by the drugsellers were listed and the number of times that each active ingredient or product name was mentioned was counted [24]. Whether the drugs dispensed to the exit customers could potentially be relevant for the symptoms/illness reported was assessed in retrospect by a general practitioner and a pharmacist. Each case was assessed individually based on reported symptoms and dispensed drugs. For example, for fever alone, antipyretics were assessed as relevant whereas antibiotics were not. Antimalarials were assessed as relevant for malaria and so forth. Further assessment criteria are available from the first author.

#### Analyses of open-ended questions

Responses to the open-ended questions were translated into English by the first author who has a fair knowledge of Swahili. She was assisted in this by native Swahili speakers with a health-related background (medical and social science) as well as a layperson. In the process of translation it became clear that the analysis must be performed using the original Swahili text in order not to lose important information. The English translations were therefore used mainly as support in discussion with the non-Swahili speaking co-authors. The material generated was analyzed using two different approaches to content analysis as described by Hsieh and Shannon [25]. Content analysis initially became renowned as a quantitative method in the field of media research where it was used to describe text data with the help of statistics [26]. Over time it has evolved into different qualitative methods that have come into wide use in health research. Qualitative content analysis focuses on the characteristics of language as communication with attention to the content or contextual meaning of the text [25].

#### Summative manifest content analysis

The respondents wrote down one sentence each to respond to the question 'Please describe what an antibiotic is'. As the depth of the material generated was limited, a summative manifest approach was deemed to be appropriate. The responses were sorted in Excel into groups according to keywords identified in the texts. The frequency of respondents using different keywords was calculated.

#### Inductive latent content analysis

The responses to the two open-ended questions: 'What is antibiotic resistance?' and 'How does antibiotic resistance occur?' were entered into the qualitative analysis software Open Code 3.4. Meaning units, i.e. parts of sentences that relate to the same central meaning, were identified and assigned codes that described the content [26]. Similar codes were thereafter grouped together to generate sub-categories and categories. One non-predefined theme emerged bottom up from the data.

## Results

### Antibiotics sale and dispensing practices

In total 350 customers from an estimated 91 drugstores in eight districts were interviewed. One third of the customers reported having seen a health worker before coming to the drugstore and almost all of these had a prescription. A majority said the health worker visited was a doctor (See Table 1). Based on the symptoms or disease reported, 83% of all dispensed drugs were assessed to be potentially relevant. Customers who did not have a prescription were sold relevant drugs more often than those buying drugs with a prescription (p = 0.048). Twenty-four percent of the respondents had bought antibiotics. The most common antibiotics bought were Flagyl (metronidazole) (n = 15), amoxycillin (n = 13) and co-trimoxazole (n = 13). Antibiotics were dispensed mainly for stomachache, cough, genital complaints and diarrhea. (See table 2) Antibiotics sold were less often assessed to be relevant than other kinds of drugs, i.e. in 51% of the cases (p < 0.000) with a non-significant difference between drugs that were not or were prescribed. Nearly all customers were told by the drugseller how to use the drug. The knowledge level of the drugseller was ranked as high or very high by 75% of the exit customers. Ninety-three percent of respondents reported they were satisfied or very satisfied with the service at the drugstore and most could afford all drugs recommended or prescribed.

**Table 1:** Selected responses to exit customer interviews performed in drugstores, duka la dawa baridi, in Tanzania.

	Number of respondents
	**(N = 350)**
	**n (%)**
The respondent reported that he/she:	
	
Had bought antibiotics (excluding SP*)	84 (24)
Bought drugs for someone else	151 (43)
Had seen health worker	104 (30)
Said that the health worker seen was a doctor	79 (23)
Had a prescription	94 (27)
Could afford all drugs prescribed/recommended	316 (90)
	
The respondent reported that he/she was:	
	
Told how to use the drug	327 (93)
Given both oral and written information	231 (66)
	
The respondent reported that he/she was informed about:	
	
The name of the drug	168 (48)
The strength of the drug	16 (5)
How many times a day to take the drug	312 (89)
The duration of treatment	220 (63)
To take drug with or without food	95 (27)
Side effects	10 (3)
Interactions with other medicines	5 (1)
Not to share drug with others	6 (2)
How to store the drug	27 (8)
To keep out of reach of children	60 (17)
	
The knowledge of the drugseller was ranked as:	
	
Very high	44 (13)
High	216 (62)
Medium	67 (19)
Low	13 (4)

*Sulfadoxine Pyrimethamine

**Table 2:** Presentation of the symptoms/illness that the exit customers reported linked to antibiotic sales.

Symptoms/Illness	Number of customers with the reported symptoms/illness	Percentage that received antibiotics	Percentage of the antibiotics bought on prescription	
	n (%)			
All	350 (100)	24		32
Malaria	95* (27)	3^k^	-	100
Cough^δ^	53 (15)	57	+	40
Stomachache^ε^	51 (15)	51	+	15
Headache^β^	38 (11)	0	-	
Fever^φ^	22 (6)	14	n.s.	0
Genital complaints	14 (4)	50	+	29
Diarrhea	10 (3)	90	+	10

(-) significantly under-represented among the customers that received antibiotics. (Fever not significant) (+) significantly over-represented. (p < 0.05)

*92 (97%) of customers with malaria received antimalarials, of these, 40% had a prescription

^κ^In addition to malaria, one of these customers also had UTI and the others had fever, headache and coughing. All received both antimalarials and antibiotics.

^δ^11% with cough also had fever (10% of those with cough who received antibiotics had fever)

^ε^14% with stomachache also had diarrhea (27% of those who received antibiotics), 4% had fever

^β ^No malaria or fever

^φ^Not reported to be malaria

### Drugseller knowledge of antibiotics

Of the 75 drug sellers who responded to the questionnaire, 64 were women. Twenty-seven were nurse-assistants, three had other health education, 17 and 16 had secondary and primary education respectively (See Table 3).

**Table 3:** Background characteristics of the 75 drugsellers responding to the antibiotic questionnaire in conjunction to an intervention in rural Tanzania.

District	A	30
	B	9
	C	36
		
Sex	Male	8
	Female	64
		
Age	Median (min-max)	28 (19-75)
		
Education	Primary	16
	Secondary	17
	Health-related	30
	Missing	12

Seventy-nine percent responded that diseases caused by bacteria can be treated with antibiotics. Of these, 24% (n = 14) also answered that it can be used for diseases caused by virus. Twelve percent stated that there is no difference in indication between different antibiotics i.e. all antibiotics can be used for treating the same diseases. Few said that headache (1%), general weakness (4%) or 'all diseases' (3%) can be treated with antibiotics whereas 85% said that STI could be treated with antibiotics. A majority of the respondents (72%) had heard of antibiotic resistance.

When asked to name some antibiotics, all participants could mention the brand or generic name of at least two products. Five also mentioned other kinds of drugs and some names could not be identified. Amoxycillin (n = 55) and Ampicillin (n = 50) were most frequently mentioned.

None of the independent variables of the multivariate logistic regression analysis had any statistically significant association with the outcome. Hench, neither age, sex, education nor district were decisive for whether the respondent knew that antibiotics treat bacterial but not viral disease.

### Keywords used to describe antibiotics

Six different keyword-groups were identified in the responses to describe what an antibiotic is: Drug that treats: 1. *Bacteria*, 2. *Infections*, 3. *Bugs/Germs*, 4. *Many diseases/*It is a *Strong drug*, 5. *Drug*, 6. Drug that treats *Resistant germs/Chronic disease *(See Table 4).

**Table 4:** Distribution of the respondents into the six different keyword-groups of the summative manifest content analysis based on responses to open ended questions in the drugseller questionnaire on antibiotics and resistance.

Keyword groups	Number of respondents n (%)	Selected quotations translated	Quotations in Swahili
*Bacteria*	20 (27)	*"Is drug that treats bacteria"*	*"Ni dawa inayotibu Bacteria"*
*Infections*	9 (12)	*"Is drug to treat infections"*	*"Ni dawa ya kutibu maambukizi"*
*Bugs/Germs*	10 (13)	*i) "Is drug that kills bugs"*	*i) "Ni dawa zinaua wadudu"*
		*ii) "Drug that can kill germs"*	*ii) "Dawa ambayo inaweza kuuwa vijidudu"*
*Many diseases/Strong drug*	12 (16)	*i) "Drug that treats more than one disease"*	*i) "Dawa inayotibu zaidi ya ugonjwa mmoja"*
		*ii) "Is strong drug"*	*ii) "Ni dawa kali"*
*Drug*	14 (19)	*"Is drug and treatment for disease"*	*"Ni dawa na tiba kwa wa magonjwa"*
*Resistant germs/Chronic disease*	4 (5)	*i) "Antibiotics is drugs that is capable of killing resistant germs in the body."*	*i) "Antibiotic ni dawa ambayo inaweza kuuwa vijidudu sugu mwilini."*
		*ii) "Is any drug that is used to treat various chronic diseases in the body."*	*ii) "Ni dawa yoyote ambayo hutumika kutibu magonjwa mbalimbali yenye usugu ndani ya mwili."*
Not interpretable or missing	6 (8)		

### Perceptions of antibiotic resistance and how it occurs

Responses to the two open-ended questions: 'What is antibiotic resistance' and 'How does antibiotic resistance occur?' gave rise to five categories, the fifth one having two sub-categories. From these, one theme emerged: 'Perceiving antibiotic resistance based on practical experience'. This theme derives from the fact that the participants seem to find the answers to these questions in what they see around them in their daily work. They give quite expected and rational descriptions of the subject from a biomedical point of view but also present less conventional explanations, possibly based on treatment failures they have witnessed. The finding that the interpretations are mainly of a practical, rather than a theoretical, nature is also based on the fact that most participants did not differentiate between the two questions and chose to give explanations of how antibiotic resistance occurs as answers to both questions. All categories hence emerged from responses to both questions. The categories and sub categories with illustrative quotations from the respondents are presented below.

#### 1. Treatment failure

This category encompasses perceptions on a microbiological level such as failure to erase or kill bacteria and the fact that the bacteria will continue to grow or rise again after treatment. The bacteria or germs are also said to be "insensitive" and "not listening" to the drug. Issues related to the drug itself and its failure to function, despite it possibly being the right drug for the disease concerned, are mentioned. Further, antibiotic resistance is described by the clinical outcome as a state where the patient is not cured by the antibiotic.

"It is that bacteria fail to die because of antibiotic" (Respondent no 32)

"There are some bacteria that are not listening to drugs." (Respondent no 4)

"Patient he/she takes the dose without feeling better or getting well." (Respondent no 55)

#### 2. Non-compliance by patients

Patients are said not to follow drug-use instructions and advice from health care providers and drugsellers. It is described how they use the drug in incomplete doses and for too short durations. They are said to forget to take their medicine or to stop taking it when they feel better.

*"Antibiotic resistance is caused by the patient himself not following the right advice given by health personnel at the dispensary or at the drugstore and not finishing the drugs given because when they feel better you will find they don*'*t continue with the drug by saying that they are cured." (Respondent no 12)*

#### 3. Provider malpractice

The patient is also described as being exposed to provider malpractice such as being given the wrong drug or the wrong dose or duration of the right drug. Additionally the issue of being given antibiotics without laboratory testing and diagnosis is discussed.

"Resistance of bacteria to antibiotics is to not be given the right dose of the concerned drug and [not] for correct time." (Respondent no 36)

"To be given drug without testing." (Respondent no 70)

#### 4. Getting used to antibiotics

It is stated that if the drug is used for a long time it ceases to work. The germs are said to get used to the antibiotic. Also the belief that the body will get used to the antibiotic if used for a long time is brought up.

"Antibiotic resistance is that maybe it [the antibiotic] has been used for a long time, therefore the body becomes used to it then if you use for other diseases it does not help you because you have used it for a long time." (Respondent no 16)

#### 5. Letting the disease linger

This category entails two sub-categories, one with a focus on waiting too long to treat a disease and one on having a disease for a long time, or having chronic disease.

i. Treatment delay

Waiting to long before treating the disease is said to make bacteria resistant. Refraining from treating a disease or a delay in seeking care is also mentioned.

"The meaning of antibiotic resistance is that there are some bacteria, even if you combine antibiotics, they are resistant because the disease has not been treated for a long time. " (Respondent no 8)

"To not take drug that was given" (Respondent no 46)

"Antibiotic resistance is caused by a patient delaying to get medical care." (Respondent no 27)

ii. Chronic disease

Antibiotic resistance and the causes are described in terms of "chronicity". Having a disease for a long time and the disease being chronic are mentioned repeatedly. Some responses are a bit confusing and it seems the respondent is trying to relate antibiotic resistance to the concept of chronic disease.

[Example of confusing answer in relation to antibiotic resistance and chronic disease]

"Antibiotic resistance is, maybe it depends on how the patient expresses himself because many patients, when they have a big problem, a serious disease, they tend to be shy to express and that is a source for the disease to be chronic". (Respondent no 10)

"Resistance of bacteria to antibiotics is because of disease staying very long time in the body, then it is hard to feel strong by medical care." (Respondent no 45)

## Discussion

To our knowledge, this is the first study from Tanzania linking antibiotic sale and dispensing practices in private drugstores to drugseller knowledge and perceptions of antibiotics and antibiotic resistance. Although the drugsellers lack higher education and formal pharmacy training, they describe the topics quite rationally from a biomedical point of view. Of all drugs dispensed, the vast majority were assessed as relevant for the symptom or disease presented. Antibiotics were significantly less often assessed as relevant compared to other drugs. The drugsellers however have a sense of which diseases can be treated with antibiotics and which cannot. What we find is probably an example of "practical knowledge", or what has been described by Schön as *knowing-in-action*[27]. Schön's theory on educating practitioners argues for upgrading the status of practical knowledge and challenges the positivistic ideas that thinking must be grounded on facts. Instead the steps of learning are described as knowing-in-action, surprise, reflection-in-action (reacting to surprise), experimentation, reflection-on-action and new knowing-in-action [28]. When educating the reflective practitioner, in this case, the "reflective drugseller", s/he must be met where s/he stands at present. The educator should be more of a coach than a teacher [27,29].

We do believe however that reflecting on one's practice without having access to scientific facts might lead to drawing unconventional or even incorrect conclusions in science-based professions such as pharmacy and medicine. The categories 'Getting used to antibiotics' and 'Letting the disease linger' are examples of this. Medical anthropology studies have shown that peoples' conceptions about medicines and health are influenced by culture and local terminology [30-32]. Haak and Hardon describe how antibiotics get "indigenized" i.e. incorporated into the local culture. Their function is described by local explanatory models; they are used in culture-specific ways and given local names [33]. In our study we found some confusion about antibiotic resistance and chronic disease. Antibiotic *resistance *in Swahili is described by the word '*usugu*'. This word however also means *chronic*. Deeper anthropological studies are needed on these matters.

The "reflective drugseller" with the perception that long-term use of antibiotics leads to resistance might be inclined to dispense short treatment courses. This practice has earlier mainly been thought to depend on the patients' limited ability to pay [34-37]. In our study, almost all exit customers could afford to buy all drugs that were recommended or prescribed. The notion that antibiotic resistance comes from not treating the patient fast enough might contribute to the practice of selling antibiotics without a formal diagnosis and prescription. Some drugsellers referred to antibiotics as a strong drug and a drug that treats many diseases, a view also found in other studies [4]. This might make drugsellers more prone to dispense antibiotics in order to achieve a successful treatment outcome even without a formal diagnosis and prescription, especially if they believe that all antibiotics can be used for the same diseases as stated by a number of the respondents.

Drugstores are only licensed to sell OTC drugs but as many as one third of the customers had a prescription which is comparable to findings of another study on private drugstores in Tanzania [37]. Having a prescription does however not guarantee that a correct diagnosis has been made by a qualified health care provider [20]. Although most of the respondents reported that the health worker they had seen was a doctor, the drugs bought on prescription were less often assessed as relevant than the drugs bought without prescription, implying low quality of the care provided by the prescriber. It is probable that the prescriber was not really a doctor, i.e. a physician, since there are very few such professionals in the rural areas represented in this study. It is also possible that customers with a prescription had more complex symptoms. The unexpected high number of prescription customers suggests that the drugstores are de facto used as 'real' (part I) pharmacies [14]. This is supported by other researchers who state that there is even a semi-formalized referral system by health care staff to drugstores. They also imply that local drugstore-inspectors might tacitly acknowledge sale of prescription-only medicines from drugstores in order not to cut off drug access in areas without pharmacies [19]. Although perhaps an intermediate solution, adjusting the regulations while turning drugstores into ADDOs can be seen as an attempt to adapt to the real situation.

The perspective in relation to antibiotic resistance, that antibiotics are common goods for health, was not investigated in our study and no drugseller brought up this matter. Perhaps the drugsellers cannot allow themselves the luxury of worrying about wider consequences of antibiotic resistance. Their challenge is instead how to best treat their customers with whichever antibiotic is available and affordable while at the same time making enough profit to sustain their business.

### Methodological considerations

The biomedical background of the researchers and data-collectors might have biased the responses towards allopathically acceptable responses. Other types of bias inherent in the different methods of analysis might however have been reduced because of method triangulation [38,39]. The study exemplifies how important the process of translation can be to data analysis. Some findings, such as the issue of chronic disease, might have been lost if the researchers performing the qualitative analyses had just been provided with translations. Further, native speakers might not reflect on particularities of their own language and this study points out the advantages of multi-cultural and multi-lingual research teams.

The number of drugsellers answering the open-ended questions was large for a qualitative study and the material used was short written statements. The regions were purposively selected and the quantitative results can therefore not be generalized to the whole of Tanzania. The results however provide a useful report of the situation in the studied regions and we find no reason to believe that they would differ markedly from other rural regions with respect to the issues studied. New private drugstores continuously open up and others close down. The exact number of drugstores in business as well as response rates are therefore hard to attain. The antibiotic questionnaire data was collected at different points in time in the different districts and in conjunction to either out-reach visits or workshops. 'District', used as a proxy for this in the multivariate logistic regression analysis, showed no statistically significant association with the outcome. The fact that the clustering effect was very small also supported that the individual respondents could be used as unit of analysis. It was not studied which kind of knowledge the drugsellers might have gained through other sources such as earlier training, other interventions or pharmaceutical representatives. The respondents' education level was however not found to be decisive for the knowledge level regarding antibiotics. The very high percentage of drugsellers answering that antibiotics can be used to treat STI might to a certain extent be due to sensitization as STI was one of the topics of the intervention although the teaching had not yet started. The assessments of the sold drugs are based on the symptoms or disease reported by the exit customer and should therefore be interpreted with some care.

## Conclusions

The drugsellers have considerable "practical knowledge" of antibiotics and a perception of antibiotic resistance based on practical experience. In the process of upgrading private drugstores and formalizing the sale of antibiotics from these outlets in resource-constrained settings, their "practical knowledge" as well as their perceptions must be taken into account in order to attain rational dispensing practices.

## Competing interests

The authors declare that they have no competing interests.

## Authors' contributions

All authors participated in project design, planning and interview guide preparation. The data was collected by research assistants supervised by PM and WK. The data analysis and manuscript preparation was performed by NV with support from PM, WK, GT and CSL. All authors approved the final version of the manuscript.

## Pre-publication history

The pre-publication history for this paper can be accessed here:

http://www.biomedcentral.com/1471-2334/10/270/prepub

## Supplementary Material

Additional file 1 **Questionnaire for Exit interviews with drugstore customers**. The English version of the questionnaire used for interviewing customers coming out from the drugstores. Click here for file

Additional file 2 **Drugseller antibiotic questionnaire**. The English version of the questionnaire on antibiotics and antibiotic resistance that was filled in by the drugsellers. Click here for file
