# Supplementary material for: "Practical knowledge" and perceptions of antibiotics and antibiotic resistance among drugsellers in Tanzanian private drugstores
Source: BMC Infect Dis. 2010 Sep 16;10:270. doi: 10.1186/1471-2334-10-270 (PMC2949758; doi:10.1186/1471-2334-10-270)
Supplement: Additional file 1 — Questionnaire for Exit interviews with drugstore customers. The English version of the questionnaire used for interviewing customers coming out from the drugstores. [file 1471-2334-10-270-S1.DOC]

Peercon project MUHAS/KI/LSHTM

# PEERCON EXIT INTERVIEW

Questionnaire Number____________________________________

To be filled in by enumerator

| 1 | Name of interviewer | 5 | Date |
| --- | --- | --- | --- |
| 2 | Name of facility | 6 | Code of Pharmacy |
| 3 | Number of interviewer | 7 | District |
| 4 | Type of facility **(Circle which one)**   1. Pharmacy (type 1 poisons) 2. Medical shops (type 2 poisons) 3. Health facility (dispensers, clinic, health centre, hospital) | 8 | Sector **(Circle which one)**   1. Private 2. Public |

**Characteristics of Respondent**

*(Interviewer read items and mark √ for every option)*

Sex of respondent

1. . .Male
2. . Female

Age of respondent __________________ (In number of years)

Education level of respondent

1. . Primary
2. . Secondary
3. . College
4. . Other formal education (eg. Madrasa)
5. . No formal education
6. . Other (specify) ___________________

### A: Dispensing practices

1. Whom did you buy the drug for?

1. Myself (go to 4i)

2. Somebody else

2. Is the patient a male or a female?

1. Male

2. Female

3. How old is the patient? __________ Years (in number of years if adult, in number of months if child less than 1)

4i. What symptoms lead you to buy medicines at this unit?

1. Itchy genitals
2. Fever
3. Headache
4. Coughing
5. Stomach ache
6. Refused to say
7. Other (specify) _____________

4ii What type of illness did you buy the drugs for?

1. Malaria
2. STI
3. HIV/
4. Diarrhoea
5. Coughing
6. Stomach ache
7. Headache
8. Fever
9. Refused to say
10. Other (specify)_________________________

5. What medicines did you buy?

_________________________________

6i Did you see a health worker before coming?

1. . Yes
2. . No (if no go to question 7)

6ii What type of health worker?

1. Doctor
2. Medical Assistant
3. Nurse
4. Any other health worker (specify) ___________________

7. Did you buy the drugs on prescription?

1. Yes (go to 9)
2. No (go to 8)

8. If not prescribed, how did you decide to buy the medicine?

1. Used it before
2. Advised by a friend/relative
3. Advised by the drug seller/pharmacist
4. I was sent to buy it by the patient
5. I know of the drug and asked for it
6. Other explanation (specify) __________________

9. Did the drug seller/pharmacists tell you how to use the medicines?

1. Yes
2. No (go to question 14)

10. How were the instructions give?

1. Orally
2. Written
3. Both written and orally

11. Did the drug seller/pharmacist ask you to repeat the instructions?

1. Yes
2. No

12. What information were you given? [Read out the alternatives and tick if respondent says yes]

| 1.The name of the product |  |
| --- | --- |
| 2.The strength of the product |  |
| 3.The dosage (how many times a day) |  |
| 4.The duration of the treatment – How many days to take this |  |
| 5.How to take it (with food, fasting etc) – To take with or without meals |  |
| 6.Side effects |  |
| 7.Interactions with other medicine |  |
| 8.How to store the product |  |
| 9. Not to share your medication with others |  |
| 10. Other (specify) |  |

13. Under what conditions did the drug seller instruct you to keep/store the remaining drugs?

1. Out of reach of children
2. In clean and dry vessels
3. In a cool and dry place
4. Out of direct sunlight

14. How do you rate the knowledge of the drug seller?

# Very high

1. High
2. Medium
3. Low

5. I do not know

**B: Accessibility and Affordability of Drugs**

15i. Were you able to afford to buy all the drugs as prescribed/advised?

a. Yes (go to 16)

b. No (go to 15ii)

15ii If No, Why?

1. I did not prefer to buy complete dose
2. I did not have enough money
3. I have the medicines at home
4. Other (specify) _________________

16. When buying drugs what determines the amount you buy?

1. The dosage prescribed
2. The amount of money I have
3. The amount advised by friends/relatives/neighbour
4. Other (specify)……………………………………..

17. Why did you decide to buy drugs from this particular outlet?

1. Near to my residence
2. Availability
3. Cheap price
4. Advised by doctor/prescriber
5. Advised by friend
6. Out let has trained personnel
7. Other (specify)……………………………………….

18. How do you rate the prices of drugs in this outlet with respect to your ability to buy them?

1. They are too expensive
2. Price are within my reach
3. I usually find them cheaper
4. I don’t know

19 (i). Do you usually compare the price of drugs before you buy?

1. Yes
2. No (go to 20)

19(ii). If Yes how do you do this?

1. Compare prices of different products within same facilities
2. Go to different facilities
3. Asked somebody
4. Other (specify) __________________________________

### C: Consumer Rights and Regulation

1. What would you do if you/a patient experiences any side effect from the drug you

buy at the outlet?

1. Go back to drug outlet
2. Go to prescriber
3. Seek medical attention elsewhere
4. Seek advice from friend/relative
5. See the drug seller
6. Complain to an authority organization (eg. TFDA
7. Take legal action
8. Don’t do anything
9. Other (specify) ______________________
10. As a drug consumer what do you consider to be your basic rights?

1. Right to know the dose of the drug

2. Right to know how to use the drug

3. To know the side effects of the drug

4. To know the price of drugs before buying

5. To se the drug seller in case anything bad happens

6. To return brought drugs

7. To return bought drugs and get the refund

8. Others (specify)____________________________

9. Do not know (go to24)

1. Have you at any point in time demanded any of the rights mentioned in 21 above?

1. Yes

2. No (go to 24)

1. If Yes which ones of the right (mention)______________________________

______________________________________________________________

1. Do you know of any organisation that protects the rights of drug users?

1. Yes

2. No (go to 26i)

3. Don’t know (go to 26i)

1. If Yes, would you please mention them

1. _______________________________

2. _______________________________

3. _______________________________

4. _______________________________

26i. Are you aware of any laws/regulations that protect drugs users/customers?

1. Yes

2. No (go to 27)

3. Don’t know (go to 27)

1. ii. If yes, please mention

1. _________________________________

2. __________________________________

3. __________________________________

4. ________________________________

1. How satisfied are you with services at this drug outlet? *(Interviewer probe how satisfied the interviewee is)*

1. Very satisfied

2. Satisfied

3. Not satisfied

4. I don’t know

28. Among the following methods, which ones do you think are the most appropriate for making people aware of consumer rights and good dispensing practices in this district?

| Methods | Rank | Consumer  Rights | Dispensing Practices |
| --- | --- | --- | --- |
| 1. Drama/ Role play |  |  |  |
| 2. Posters |  |  |  |
| 3. Leaflets |  |  |  |
| 4. Traditional dancing/ngoma |  |  |  |
| 5. Seminar/Workshops |  |  |  |
| 6. Printed garments (T-shirts and caps) |  |  |  |
| 7. Social groups (peer or colleague groups) |  |  |  |
| 8. Radio programmes |  |  |  |
| 9. Other (specify) |  |  |  |

29. Have you ever thought of learning about drug consumer rights?

1.Yes

2. No

30. Would you be interested in getting further education concerning drug consumer rights?

1. Yes

2. No

3. I don’t know

31. Who would be the right people in this district you would believe most if delivering messages related to drug consumer rights?

1. Health worker

2. Lawyer

3. Head of Faith Based Organization (FBOs)

4. Drug seller

5. Others (specify) ________________

6. I do not know/not sure

32. Are any of the following a form of malpractice?

1. Giving a wrong dose or dosage

2. Dispensing prescription drugs without prescription

3. Giving drugs without proper information

4. Changing prescriptions without the prescriber’s consent

## What information do you expect to get from a drug store?

1. Information on general health

2. Information on drugs

3. Information on diseases

4. Information in taking drugs

5. How to prevent disease

6. Side effects

7. Other__________________

8. I do not know

## Thank you
