# Supplementary material for: "Practical knowledge" and perceptions of antibiotics and antibiotic resistance among drugsellers in Tanzanian private drugstores
Source: BMC Infect Dis. 2010 Sep 16;10:270. doi: 10.1186/1471-2334-10-270 (PMC2949758; doi:10.1186/1471-2334-10-270)
Supplement: Additional file 2 — Drugseller antibiotic questionnaire. The English version of the questionnaire on antibiotics and antibiotic resistance that was filled in by the drugsellers. [file 1471-2334-10-270-S2.DOC]

Peercon project MUHAS/KI/LSHTM

1. Questionnaire number________________
2. District____________________________
3. Place of interview___________________

Questionnaire to be filled in at the beginning of the STI session.

1. Date:_____________________________
2. Age (in years): ____________________
3. Sex: □ Male □ Female
4. Educational background: ______________________________________________
5. Please describe what an antibiotic is:

____________________________________________________________________________________________________________________________________________________________________________________________________________________________________________________________________________________

1. Please mention some different antibiotics that you know:

____________________________________________________________________________________________________________________________________________________________________________________________________________________________________________________________________________________

1. Please tick which of the following can be treated with antibiotics:
   1. All diseases □ Yes □ No □ I don’t know
   2. Headache □ Yes □ No □ I don’t know
   3. Bacterial diseases □ Yes □ No □ I don’t know
   4. Viral diseases □ Yes □ No □ I don’t know
   5. Thrush □ Yes □ No □ I don’t know
   6. Candida □ Yes □ No □ I don’t know
   7. Sexually transmitted -

diseases □ Yes □ No □ I don’t know

- 1. General weakness □ Yes □ No □ I don’t know

1. Can all antibiotics be used for the same diseases?

□ Yes □ No □ I don’t know

1. Have you heard of antibiotic resistance?

□ Yes □ No □ I don’t know

1. What is antibiotic resistance?

______________________________________________________________________________________________________________________________________________________________________________________________________________________________________________________________________________________________________________________________________________________________________________________________________________________________________________________________________________

1. How does antibiotic resistance occur?

______________________________________________________________________________________________________________________________________________________________________________________________________________________________________________________________________________________________________________________________________________________________________________________________________________________________________________________________________________
